# Clinician‐interpreter collaboration in multilingual dementia assessments: Barriers, role boundaries, and clinician training needs

**DOI:** 10.1002/dad2.70515

**Published:** 2026-10-20

**Authors:** Marina Cavuoto, Nyssa Clarke, Simona Markusevska, Erika Gonzalez Garcia, Jim Hlavac, Lee‐Fay Low, Robyn Woodward‐Kron, Josefine Antoniades, Joanne Enticott, Andrew Simon Gilbert, Dina LoGiudice, Xiaoping Lin, Kerry Hwang, Jennifer White, Bianca Brijnath

**Affiliations:** ^1^ School of Humanities and Social Sciences La Trobe University Bundoora Victoria Australia; ^2^ Division of Social Gerontology National Ageing Research Institute Parkville Victoria Australia; ^3^ School of Global, Urban and Social Studies RMIT University Melbourne Victoria Australia; ^4^ School of Languages, Literatures, Cultures and Linguistics Monash University Clayton Victoria Australia; ^5^ Faculty of Medicine and Health University of Sydney Sydney New South Wales Australia; ^6^ Faculty of Medicine Dentistry & Health Sciences University of Melbourne Parkville Victoria Australia; ^7^ Faculty of Medicine, Nursing and Health Sciences Monash University Clayton Victoria Australia; ^8^ School of Health and Social Development Deakin University Burwood Victoria Australia; ^9^ Department of Medicine Royal Melbourne Hospital University of Melbourne Parkville Victoria Australia; ^10^ Hunter New England Local Health District Newcastle New South Wales Australia

**Keywords:** clinicians, cognitive assessment, dementia, intercultural communication, interpreters, training

## Abstract

**INTRODUCTION:**

Clinicians often work with interpreters when assessing multilingual patients for dementia. We examined factors shaping clinician‐interpreter communication and which aspects of clinician practice could be strengthened through training.

**METHODS:**

We interviewed 16 clinicians and 24 interpreters involved in cognitive assessments across Australia between May 7 and December 9, 2024. Data were analyzed using the framework approach.

**RESULTS:**

Clinicians valued interpreters’ expertise beyond language translation, including identifying language cues (e.g., pronunciation or word‐finding difficulties) and mediating across cultures. In turn, they facilitated patients’ and families’ acceptance of interpreters. However, booking processes, interpreting modes (e.g., telephone interpreting), unclear role boundaries, and time constraints (especially for briefings and debriefings) limited communication quality. Both groups indicated that clinician training was needed to support culture‐fair assessment and include interpreters as part of the care team.

**DISCUSSION:**

Training clinicians to work effectively with interpreters may improve diagnostic accuracy and care for multilingual patients with dementia.

## Highlights

- Interviews with 40 clinicians and interpreters in Australia found that booking processes, interpreting modes, unclear role boundaries, and time constraints limited clinician‐interpreter communication quality.
- Training clinicians can support culture‐fair assessment.
- Including interpreters as part of the healthcare team may improve timely and accurate diagnosis for multilingual patients.

## 1 BACKGROUND

Aphasia, impaired working memory, and impaired executive function are dementia symptoms affecting communication. Those who are multilingual may lose their second and subsequent languages before they lose proficiency in their native language.^1^ Losing proficiency in the dominant language of one's place of residence can impede patient‐clinician communication. Such patients are less likely to advocate for themselves and ask questions about their diagnosis and treatment, more likely to have limited interactions and updates from clinical staff, and feel excluded from decision‐making.^2^, ^3^ Clinicians, in turn, may compensate through overuse of imaging and pathology investigations,^2^ unnecessary hospital admissions, and longer hospital stays, which is costly to the health system and undermines the quality of patient care.^4^, ^5^

Collectively, these barriers delay timely and accurate diagnosis. This study was undertaken in Australia, where English is the dominant language and dementia the leading cause of death, disproportionately affecting ethnically diverse communities.^6^ About one in five Australians aged ≥65 years were born in non‐English‐speaking countries, and 18% speak a language other than English at home.^7^ Of over 200 languages spoken, Italian, Mandarin, Cantonese, and Greek are the most common non‐English ones.^7^ With cohort flow and shifting migration patterns from Europe to Asia and Sub‐Saharan Africa, Australia's older population will become larger and more ethnically diverse by 2056.^8^

Australian dementia registry data show that older people from non‐English‐speaking backgrounds wait approximately 2 months from their first appointment to receive a formal dementia diagnosis, whereas White, English‐speaking Australians wait less than a month.^9^ This diagnostic disparity can be reduced by utilizing professional interpreters. During cognitive assessments, interpreters can shape the linguistic interplay between patients and clinicians thereby facilitating timely diagnosis, access to treatment, and postdiagnostic supports.^10^, ^11^ One factor that influences the effectiveness of an interpreter‐mediated cognitive assessment is the clinician's preparedness to work with interpreters. Clinicians who are trained on the impact of language barriers in healthcare are better equipped to address cross‐cultural issues that may arise in linguistically discordant consultations and achieve better patient outcomes.^12^, ^13^, ^14^ Untrained or insufficiently trained clinicians may have limited confidence in how to work effectively with interpreters, not know how to lead interactions, how to brief and debrief interpreters, or how to form a three‐way therapeutic alliance with the patient and the interpreter.^11^

Apart from training, the other factors that can influence effective intercultural communication between clinicians and interpreters during a cognitive assessment remain largely unknown. Addressing this lacuna, we investigate the factors shaping effective intercultural clinician‐interpreter communication and which aspects of clinician practice could be strengthened through training.

**RESEARCH IN CONTEXT**

1. **Systematic review**: We reviewed the literature using traditional sources (e.g., PubMed) as well as meeting abstracts and presentations. Using professional interpreters does improve timely and accurate dementia diagnosis for multilingual patients. However, the factors that influence effective intercultural communication between clinicians and interpreters during cognitive assessment remain largely unknown.
2. **Interpretation**: Our interviews with 40 clinicians and interpreters showed that although clinicians appreciated professional interpreters, knowledge gaps hindered clinicians from working effectively with interpreters.
3. **Future directions**: Clinician training should focus on how culture and language influence dementia and cognitive assessments, provide strategies to minimize bias in intercultural cognitive assessments, guide clinicians in leading briefings and debriefings, and support the application of clinical judgment alongside culture‐fair assessment tools to enable accurate diagnosis. Training should be online, self‐paced, and under 90 min.

## 2 METHODS

### 2.1 Design

A qualitative descriptive design was applied^15^ using interview data from clinicians and interpreters participating in the Improving Interpreting for Dementia Assessments (MINDSET) study. MINDSET focused on training interpreters and the study's research design,^16^ training content,^17^ effectiveness,^18^ and implementation^19^ are published. Ethical approval was provided by the University of Western Australia Human Research Ethics Committee (HREC#2021‐0477). This study is reported using the Consolidated Criteria for Reporting Qualitative Studies.^20^

### 2.2 Recruitment

We recruited clinicians if they worked (i) in health settings offering diagnostic dementia services (e.g., memory clinics, hospitals, private clinics) and (ii) with interpreters during cognitive assessments for dementia at least once a month. To ensure national coverage and a mix of different clinicians (e.g., geriatrician, neuropsychologist), we diversified our sampling framework by state and profession. Through purposive and snowball sampling, we sent emails to individual clinicians and dementia diagnostic services working in catchment areas with high linguistic diversity notifying them about the study and asking eligible clinicians to email an expression of interest. Twenty‐three responded, of whom 16 were interviewed (the remaining seven did not respond to further correspondence or were unavailable).

Similarly, we developed a purposive sampling framework to ensure diversity across sex, language, geographical location (state and urbanicity), and credentialled status for interpreters. Of 768 interpreters enrolled in the MINDSET training, 140 were shortlisted and invited for an interview. We aimed for a sample of 20 clinicians and interpreters each but stopped at 16 and 24 respectively when we reached data saturation.^21^

### 2.3 Procedures

Following written informed consent, interviews were conducted one on one, online via Microsoft Teams, between May 7, 2024 and December 9, 2024. Interviews were conducted by NC, SM, or MC, female researchers who have conducted research related to health services, trauma, and older people. MC is a clinical neuropsychologist. Questions focused on understanding barriers experienced by clinicians and interpreters during dementia assessments, their role boundaries, and what, if any, areas needed to be improved through clinician training (Supplementary file 1). Interview questions were based on interpreter and clinician feedback during the codesign workshops,^17^ the extant literature,^22^, ^23^, ^24^, ^25^ and the Reach, Effectiveness, Adoption, Implementation, and Maintenance (RE‐AIM) framework, which informed the training's implementation.^19^ The interview guide was not pilot tested, and participants were not provided with a copy of the questions prior to interview. Interviews were ∼60 min in duration, in English, and audio recorded. Participants were remunerated at their average hourly professional rate. Repeat interviews were not conducted.

### 2.4 Analysis

Audio recordings were professionally transcribed, then identifying data were removed. Using the framework approach,^26^ NC and BB coded the same two transcripts independently and then met to discuss codes. A working schema was developed and reapplied by NC and MC on two additional transcripts, resulting in further refinements and developing a draft analytical framework. The cycle was repeated twice more with NC, SM, and BB before the analytical framework was finalized. NC then coded all transcripts in NVivo (version 12.6.0.959). We kept a record of the codes and themes through this process to trace how our thinking and data interpretation developed. This iterative approach ensured continuous negotiation and consensus building between the researchers.^27^ The themes and interpretations were also presented to the wider team, including clinicians (DG, L‐FL, JW), interpreters (JH, EGG), and health and social care researchers (JE, KH, RW‐K, JA, AG) for verification and refinement. An audit trail, clear analytic process, and peer debriefing enhanced rigor and replicability.^27^ Credibility was achieved through reflexivity and transparency through team discussion and the use of verbatim quotes.^27^ Data triangulation was ensured through comparing different participant groups (interpreters and clinicians), multiple investigators analyzing the data, and data interpretation being informed by diverse professional perspectives (e.g., clinicians, interpreters) and knowledge sources (e.g., patient‐clinician communication, clinical training pedagogy, cognitive assessment). Thus, through triangulation, audit trails, and consensus validation (Figure 1), we established data trustworthiness.^27^

**FIGURE 1:**
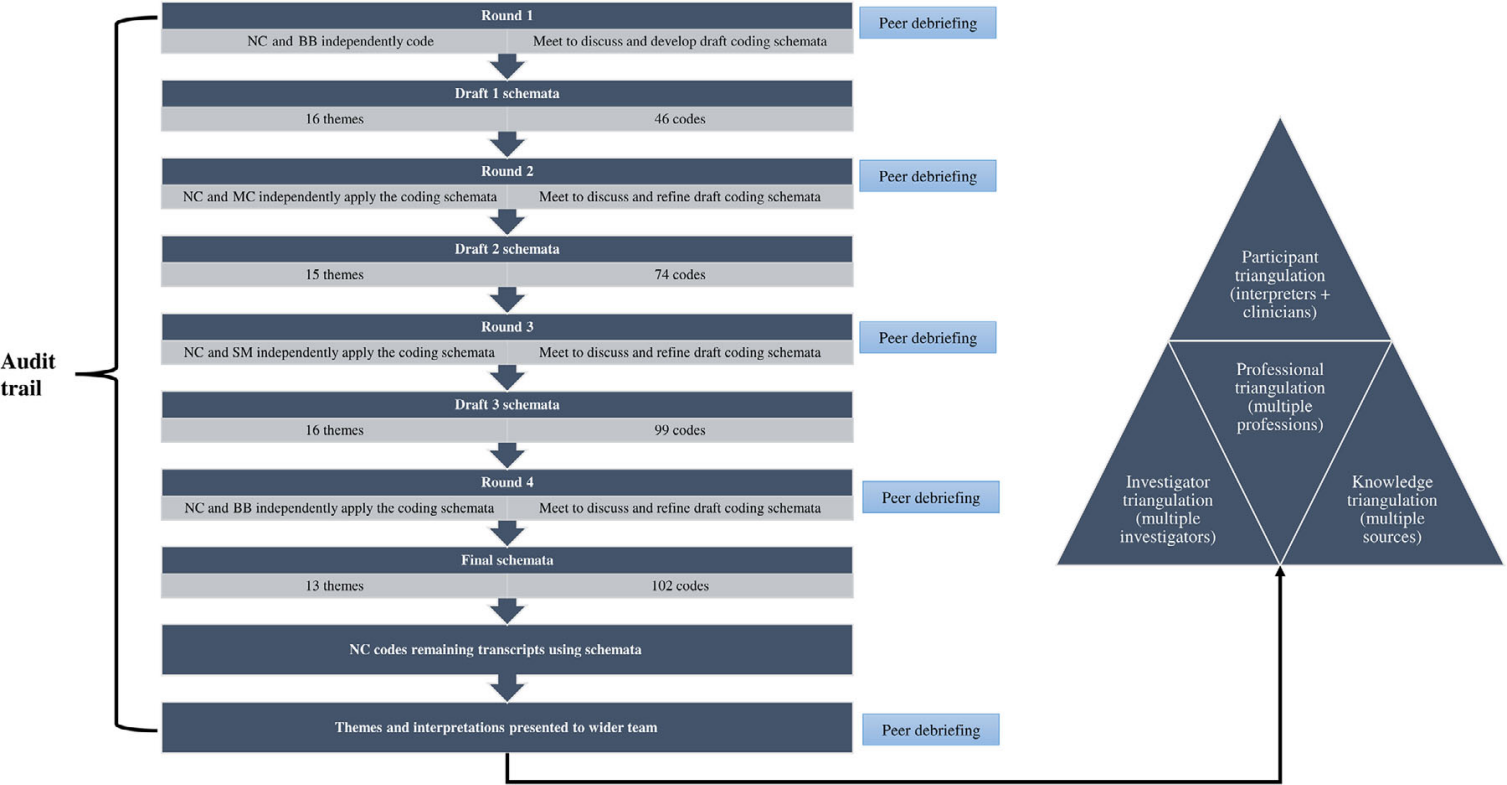
Data analysis process.

## 3 RESULTS

Table 1 describes participant characteristics. Interpreters worked across a variety of linguistic backgrounds: European languages (e.g., Italian) were the most frequently represented (7, 29.2%), followed by Middle Eastern (e.g., Arabic) (6, 25.0%), East Asian (e.g., Mandarin) (4, 16.7%), and Auslan users (3, 12.5%). There were two (8.3%) interpreters in South Asian languages (e.g., Hindi) and one (4.2%) each in African (e.g., Amharic) and Southeast Asian (e.g., Vietnamese) languages.

**TABLE 1:** Participant characteristics.

Characteristics	Interpreters	Clinicians
	***n* (%)**	
Sex female *n* (%)	18 (75%)	12 (75%)
Urban‐based	20 (83%)	16 (100%)^a^
State		
Australian Capital Territory	1 (4%)	0
New South Wales	6 (25%)	2 (13%)
Northern Territory	0	0
Queensland	1 (4%)	1 (6%)
South Australia	4(17%)	0
Tasmania	2(8%)	0
Victoria	7(29%)	13(81%)
Western Australia	3(13%)	0
Years of experience in role^b^ (median)	19	12.5

^a^ One clinician reported working in both an urban and regional setting.

^b^ Years of experience in role refers to years of interpreting experience for interpreters and clinical dementia assessment experience for clinicians.

Clinicians included geriatricians (6, 37.5%), clinical neuropsychologists (4, 25%), social workers (3, 18.8%), a physiotherapist (1, 6.3%), a psychiatrist (1, 6.3%), and an occupational therapist (1, 6.3%). Eight participants (50%) worked in public health settings, two (12.5%) in private settings, and six (37.5%) across both. Four participants (25%) were academic clinicians holding conjoint university appointments. Clinicians’ frequency of cognitive assessments with interpreters ranged from once per month to three times per week. They commented positively on interpreters, acknowledging the latter's skillsets extended beyond multilinguistic fluency to critical communication skills, such as conveying language cues to facilitate diagnostic assessment (e.g., pronunciation difficulties, word‐finding difficulties, and difficulty with comprehension) and mediating across diverse cultures.

“It [interpreting encounter] can be a really positive experience. If you've got someone who's very anxious about being assessed, especially if words like ‘dementia,’ ‘aged care homes’ and things like that are on the table, having someone who is warm, speaks their language, can really make them more at ease during the assessment” (*Clinician #16, woman, social worker, 10 years of experience*).

Simultaneously, participants identified obstacles to effective intercultural communication: (i) the booking process and interpreting mode, (ii) rejection of the interpreter by the patient and their family, (iii) confusion on role boundaries, and (iv) time constraints. Improving clinicians’ interaction with interpreters through further training emerged as a strong inductive finding highlighted by both groups. Topics listed for further clinician learning included recognizing the interpreter as part of the healthcare team and making cognitive assessments more culturally fair. We expand on these findings below.

### 3.1 Booking process and interpreting mode

How interpreters were booked and the interpretation mode (e.g., face‐to‐face, telephone) shaped effective clinician‐interpreter communication. When clinicians were able to assess the linguistic needs of their patients and book an interpreter, they reported feeling confident about the process. In contrast, when patients’ linguistic needs were assessed by hospital administrative staff, clinicians felt they had reduced opportunity to input into the interpreting needs of the patient or note additional complexities that might need to be considered.

“If there's specific needs for the patient – it needs to be a female interpreter or if there's a specific dialect – sometimes that might not even be made clear in the patient's file. Stuff like that can make it difficult when they rock up and it's the wrong interpreter” (*Clinician #8, woman, clinical neuropsychologist, 10 years of experience*).

Clinicians consistently reported that the availability, flexibility, skill, and professionalism of in‐house interpreters (e.g., those working solely in a hospital) were higher compared to interpreters sourced through external agencies (e.g., those working across multiple settings outside healthcare). Externally contracted interpreters had limited opportunities to engage with clinicians to receive background information at the time of the booking. If they practiced in languages with high demand but limited supply of interpreters, they were usually busy and had less time for training.

“Interpreter availability has really declined since the pandemic and also as the pool of interpreters has aged. There are fewer interpreters for particular languages … like Greek and Italian, which used to be very readily available” (*Clinician #6, woman, clinical neuropsychologist, 25 years of experience*).

Clinicians and interpreters also reported that the mode of interpreting (e.g., telephone, videoconference, in‐person) influenced the quality of cognitive assessments. Telephone interpreting, increasingly used following the COVID‐19 pandemic, was recognized as more time‐ and cost‐effective compared to videoconferencing or face‐to‐face interpreting. However, participants noted that telephone interpreting disadvantaged patients with cognitive and/or hearing impairments as interpreters could not observe body language or visual cues from either the clinician or the patient.

“You've got a cognitively impaired person who's often hearing impaired, having difficulty with the telephone, not understanding even what we're trying to achieve, being asked what seems to be stupid questions. There's a great disadvantage we put upon the recipient of the test” (*Interpreter #986, man, certified provisional interpreter, Italian, 30 years of experience*).

### 3.2 Facilitating acceptance of interpreter's presence

Communication quality was affected by whether the patient and family accepted the interpreter's presence during the assessment and the clinician's ability to manage conflict if they did not. Challenges to the interpreter's presence sometimes occurred, for example, family members criticizing the interpreter's work, answering on the patient's behalf, telling the interpreters that they were not needed, asking them to leave, or stepping into the interpreting role. Interpreters expected clinicians to intercede on their behalf as they were leading the assessment. Clinicians reported being confident in their ability to manage such conflict, but interpreters were more equivocal.

“It is a problem. They [family members] do interrupt and they do start talking for the patient. So, the professional [clinician] needs to stop them. We can't stop them. We stop them, they get really mad with us” (*Interpreter #349, man, certified interpreter, Sinhalese, 20 years of experience*).

“The family, you could tell straight away, were not happy with the … interpretation. So, I just aborted it. And just reverted to the family as translators” (*Clinician #1, man, geriatrician, 5 years of experience)*.

### 3.3 Confusion on role boundaries

There was confusion about both groups’ professional boundaries. Some clinicians were uncertain and distrusting of the accuracy of the information conveyed when (i) interpreters appeared to say much more or less than the clinician, (ii) the interpreter's explanation to the clinician of what was conveyed was slightly different from what the clinician stated, or (iii) when the clinician became aware that the interpreter was providing additional prompts.

“Sometimes you get the sense that not everything's being translated … I give a short kind of one sentence, and it goes on quite a while …” (*Clinician #1, man, geriatrician, 5 years of experience*).

As this quote highlights, clinicians’ difficulty in navigating such scenarios stemmed from limited clarity about what was being conveyed, apprehension regarding cultural sensitivities, and reluctance to be perceived to be “telling them [interpreters] how to do their job.” Gender influenced the clinician‐interpreter dynamic:

“Sometimes if they're males, they might actually just have a little bit more of an exchange with the patient without actually telling me what that content is. And I know that it's over and above what I have stated in my question to the patient” (*Clinician #5, woman, clinical neuropsychologist, 20 years of experience*).

By contrast, some clinicians found it helpful when interpreters conveyed observations about the patient (e.g., confusion, tangentiality) and commented on changes in the patient's cognitive status if they had previously worked with them. Some clinicians also let interpreters administer cognitive assessments with limited supervision if the latter were sufficiently experienced.

“Some experienced interpreters have already done the RUDAS [Rowland Universal Dementia Assessment Scale], our typical screening test, for so long, they can even remember all the questions themselves. So, they don't need to wait for us to ask a question, then translate, and then they tell the client. They do that themselves. To me it's like it is time saving” (*Clinician #22, woman, aged care assessor, 1.5 years of experience*).

However, most interpreters reported discomfort when they were asked by clinicians to lead the assessment or administer a test (e.g., RUDAS), rather than interpreting what the clinician had stated.

### 3.4 Time constraints

The additional time required for interpreter‐mediated cognitive assessments was a significant pressure point for clinicians and interpreters. Booking extended‐length appointments could increase waitlist times and compromise service funding. Decisions about allocating additional time were made by health service managers, and time constraints could lead to clinicians skipping pre‐session briefings and interpreters feeling unable to request them, reduced opportunities for interpreters to introduce themselves, less time for clinicians and interpreters to build rapport with patients, and a tendency to rush or omit parts of the assessment.

“Pre‐interview discussion and post‐interview feedback and discussion, it's important … because of time, health professionals, they don't do that” (*Interpreter #1828, man, recognized practicing interpreter, Macedonian, 45 years of experience*).

“I would definitely recommend that the clinician has a pre‐meeting with the interpreter. Explain very briefly what their meeting is about, rather than just walking in … If you say, ‘We're about to tell Mr. Jones he has dementia and he has very poor understanding and he can get quite agitated,’ that really informs the interpreter” (*Clinician #16, woman, social worker, 10 years of experience*).

Both groups recognized the value of briefings and debriefings. Interpreters reported that these sessions helped them feel prepared, provided space to raise culturally relevant considerations, and created opportunities to reflect on challenges, learn from mistakes, and strengthen collaborative working relationships for future assessments. Clinicians said that an open and supportive dialogue during the pre‐assessment briefing enabled them to gauge the interpreter's level of experience and determine the type and amount of contextual information they needed to share.

### 3.5 Clinician training

Clinicians were positive about receiving training on working with interpreters. Some indicated that this would be something they would take up themselves, while others stated that they would recommend it for those less experienced. Suggested time frames were mostly 30 to 90 min, and it was further recommended that the training be self‐paced, online, or otherwise on‐site during lunch grand rounds with the possibility for continuing professional development points.

“I'd happily undertake the training … If it's been prepared in a collaborative way with interpreters, it would be helpful for us to know better how to work with interpreters” (*Clinician #6, woman, clinical neuropsychologist, 25 years of experience*).

Clinicians wanted to know more about the interpreter's role, how to optimize communication with interpreters, what they could ask them, and how much background information they needed. Interpreters noted that such training could also help clarify expectations, particularly where clinicians might expect interpreters to lead communication in ways that fall outside their professional scope. They also emphasized that clinicians play an important role in reassuring patients about the interpreter's presence during consultations by explaining that interpreters are bound by confidentiality under a professional code of conduct.

Both groups noted the lack of cultural appropriateness of certain tests and questionnaires and felt clinicians needed to receive training on how culture influenced assessment tools. While it was expected that clinicians would lead assessments, it was unclear how and when the interpreter should adapt questions to make them more acceptable and understood.

“You can still get a valid assessment by listening to what an interpreter says about the inappropriateness of certain stimuli and just looking at things more qualitatively rather than quantitatively” (*Clinician #5, woman, clinical neuropsychologist, 20 years of experience*).

Clinicians also wanted to learn more about providing culturally appropriate care, for example, addressing patients respectfully, managing dynamics with interpreters and patients, and delivering diagnoses in a culturally appropriate way.

“How the word ‘dementia’ is translated in the language … sometimes it's translated as a medical term and sometimes it's more like ‘crazy’ or ‘insane’ … some feedback from the interpreter about how in that culture the word is used and what's the best way to describe this” (*Clinician #23, woman, occupational therapist, 20 years of experience*).

Finally, both groups acknowledged that respecting each professional's expertise strengthened collaboration and the likelihood of obtaining a valid cognitive assessment. Though integrating interpreters as members of the healthcare team was challenging given the brief and episodic nature of the collaboration, both parties indicated that they needed training to clarify roles and enhance the quality and integrity of interpreter‐mediated cognitive assessments.

## 4 DISCUSSION

Clinicians in our study valued the role of interpreters during cognitive assessments for dementia. Echoing previous systematic reviews,^14^, ^24^, ^28^ our participants said that using interpreters improved patient‐clinician communication, reduced errors and misunderstanding, increased appropriate healthcare utilization, and led to better clinical outcomes. US studies^29^, ^30^ have also found a preference for in‐person interpreting and briefings and debriefings, despite the time constraints faced in clinical practice. Clinicians value interpreters’ linguistic skills and their capacity to broker cultural understandings of medical conditions such as dementia, its associated symptoms, and care pathways, including residential aged care; this cultural mediation was likened to “emotional work” that could help alleviate the stigma associated with dementia in many communities, increase the acceptability of seeking help from formal services, and reduce patient and family concerns.^29^, ^31^

We also found that the gender dynamic between interpreters and clinicians could affect their interactions. Interpreters’ gender can shape how comfortable patients feel, the development of trust, and clinician‐patient satisfaction in the clinical interaction, especially if the topics are sensitive and/or gender‐specific (e.g., matters to do with sexual and reproductive health or family violence).^32^, ^33^, ^34^ Female interpreters may face sexism when undertaking health interpreting; a South Korean study^32^ showed female interpreters had to perform aesthetic labor in addition to language work to meet the requirements of a competitive and oversaturated language market. Our work showed that gender and experience intersected during the clinical interaction and could create confusion about role boundaries, especially when male interpreters exceeded their scope of practice and female clinicians were compelled to reinforce role boundaries.

Working with interpreters requires advanced communication skills,^25^ and clinicians recognized that they would benefit from structured training. Training effectiveness is increased when the program is codesigned with clinicians and interpreters and includes practical skill development, interactive activities, and clinical skills assessment.^13^, ^23^ Specific to dementia, our participants thought such training would help clinicians better understand how culture and language influence dementia and cognitive assessments, lead to strategies to minimize bias in intercultural cognitive assessments, lead briefings and debriefings, and apply clinical judgment alongside culture‐fair assessment tools to support accurate diagnosis. Studies have shown that such training improves clinician knowledge,^22^, ^23^ confidence,^35^ and skills.^25^, ^36^

Departing from previous studies^13^, ^25^ that prioritized in‐person learning over several days or weeks, our participants preferred online training delivered in under 90 min. This discrepancy is likely due to the fact that most training interventions in this area were published before 2019 when online learning had fewer engagement features, was unfamiliar to learners, and had less uptake. Additionally, most training interventions have been conducted in academic environments such as teaching hospitals, medical schools, and academic conferences.^13^ In contrast, our participants were busy clinicians working in the post‐COVID era in public and private facilities, which were not all teaching hospitals or affiliated with university medical schools. Their preference for short online self‐paced training likely reflected their heavy workloads, limited time, and, if in private practice, reduced access to in‐house interpreters and peer learning opportunities such as grand rounds. Time constraints^37^, ^38^ and the absence of clear workplace policies and procedures to support consistent and standardized practice^4^ have been shown to impede clinician and interpreter communication. These organizational and systemic barriers cannot be mitigated by workforce training alone. For example, an Australian study^39^ found that, although clinicians reported increased knowledge and confidence in working with interpreters 2 months after training, their actual clinical practice did not change.

Moreover, there are unequal power dynamics between the two professions, and although clinician training might create a more balanced power relationship between clinicians and interpreters, it will not equalize it (nor should it). Interpreters and clinicians expect the latter to lead cognitive assessments. Collaborative professional exchanges between the groups can enhance cross‐cultural care,^14^, ^24^, ^28^, ^31^ but this is more likely in public hospital and medical school settings that serve a large multilingual patient population and employ in‐house interpreters as opposed to private practices staffed by small health teams. US studies^29^, ^40^ show that the foundations of the relationship are strengthened when interpreters feel a sense of belonging, often created when they and clinicians are both employed by the same organization and routinely work together (unlike external agency‐contracted interpreters, who work on an ad hoc basis with clinicians).

Finally, training can strengthen clinicians’ confidence in mediating when patients and family members do not accept the interpreter's presence and disrupt the assessment, making accurate interpreting difficult. While interpreters expected clinicians, as leaders of the assessment, to manage these situations, clinicians’ intervention was not always consistent. Training can help clinicians establish expectations at the start of interactions, alleviate the burden on family members to act as interpreters, and help clinicians maintain greater control over the interaction. This may help preserve the structure required for accurate interpreting while ensuring that the patient remains central to the assessment.

### 4.1 Limitations

Our clinician participants were predominantly from the state of Victoria and practiced in urban areas. While this aligns with where most of Australia's multilingual populations reside,^41^ our sample excluded perspectives from other states and rural areas. Our inclusion criteria that clinicians must work with interpreters during cognitive assessments at least once per month may have also resulted in our recruiting a sample that might have been particularly motivated and skilled in working with interpreters, which may not be reflective of the broader clinician workforce. Finally, we did not interview multilingual patients or family carers who might have had differing views about the quality of interpreter‐mediated communication. Their interactions with interpreters during cognitive assessments are often limited to a single encounter, and patients and families frequently describe this stage of the diagnostic process as stressful and burdensome.^42^

## 5 CONCLUSION

In multilingual aging nations like Australia, clinicians need to work with interpreters to assess patients with limited English proficiency.^10^ Without training, clinicians may rely on unqualified staff or family members, risking miscommunication and emotional burden on families. Training addresses this gap, equipping clinicians to work effectively with interpreters, thereby improving communication, avoiding harmful practices, and increasing timely and accurate diagnosis for multilingual patients.

## AUTHOR CONTRIBUTIONS

The concept and design of the study were developed by Brijnath, Enticott, Gilbert, Gonzalez, Hlavac, Low, LoGiudice, Woodward‐Kron, Antoniades, Hwang, and White. Data acquisition, analysis, and interpretation were undertaken by Brijnath, Clarke, Markusevska, and Cavuoto. The manuscript was drafted by Cavuoto and Brijnath and critically reviewed for important intellectual content by Clarke, Markusevska, Enticott, Gilbert, Gonzalez, Hlavac, Low, LoGiudice, Antoniades, Lin, White, and Hwang. All authors read and approved the final manuscript. All correspondence about the manuscript should be addressed to Bianca Brijnath, La Trobe University, Melbourne, Australia 3086. Email: b.brijnath@latrobe.edu.au

## CONFLICT OF INTEREST STATEMENT

EGG reports receiving grant funding to RMIT University from the Australian National Health and Medical Research Council (NHMRC), administered by the National Ageing Research Institute, to complete interpreter testing and remunerate the assessors who evaluated the performance of the interpreters during the conduct of the study. At the time the grant was awarded, the Australian Institute of Interpreters and Translators (AUSIT) was one of the research partners, and EGG was the president of this professional association; the AUSIT also provided in‐kind support for the project. L‐FL reported receiving grants from the NHMRC and Medical Research Future Fund, non‐financial support from Dementia Alliance International and ADNeT, and personal fees from Roche outside the submitted work. No other disclosures were reported. Author disclosures are available in the Supporting Information.

## CONSENT STATEMENT

All human subjects provided informed consent.

## Supporting information



Supporting Information

Supporting Information
