# Supplementary material for: Clinician‐interpreter collaboration in multilingual dementia assessments: Barriers, role boundaries, and clinician training needs
Source: Alzheimers Dement (Amst). 2026 Oct 20;18(4):e70515. doi: 10.1002/dad2.70515 (PMC13683551; doi:10.1002/dad2.70515)
Supplement: Supplementary file 1 [file DAD2-18-e70515-s002.pdf]

ICMJE DISCLOSURE FORM

Date: 7/31/2026

Your Name: Josefine Antoniades

Manuscript Title: Clinician-interpreter collaboration in multilingual dementia assessments: Barriers, role boundaries, and clinician training needs

Manuscript Number (if known): DADM-D-26-00213

In the interest of transparency, we ask you to disclose all relationships/activities/interests listed below that are related to the content of your manuscript. "Related" means any relation with for-profit or not-for-profit third parties whose interests may be affected by the content of the manuscript. Disclosure represents a commitment to transparency and does not necessarily indicate a bias. If you are in doubt about whether to list a relationship/activity/interest, it is preferable that you do so.

The author's relationships/activities/interests should be defined broadly. For example, if your manuscript pertains to the epidemiology of hypertension, you should declare all relationships with manufacturers of antihypertensive medication, even if that medication is not mentioned in the manuscript.

In item #1 below, report all support for the work reported in this manuscript without time limit. For all other items, the time frame for disclosure is the past 36 months.

		Name all entities with whom you have this relationship or indicate none (add rows as needed)	Specifications/Comments (e.g., if payments were made to you or to your institution)
**Time frame: Since the initial planning of the work**			
**1**	All support for the present manuscript (e.g., funding, provision of study materials, medical writing, article processing charges, etc.) **No time limit for this item.**	☐ **None**	
		National Health and Medical Research Council Australia	Grant funding to the National Ageing Research Institute (NARI)
		NAATI — National Accreditation Authority for Translators and Interpreters	Grant and in-kind funding to NARI
		AUSIT — Australian Institute of Interpreters and Translators	In-kind funding to the project
		Dementia Australia	In-kind funding to the project
		New South Wales Health Care Interpreting Service	In-kind funding to the project
		All Graduates Interpreting and Translation Services	Grant and in-kind funding to NARI
		Australian Government Department of Home Affairs Translation and Interpreting Service	In-kind funding to the project
		Australian Government Department of Health, Disability, and Ageing	Grant funding to NARI
		Televic	In-kind funding to the project
		Migrant and Refugee Health Partnership	In-kind funding to the project
**Time frame: past 36 months**			
**2**	Grants or contracts from any entity (if not	☒ **None**	

		Name all entities with whom you have this relationship or indicate none (add rows as needed)	Specifications/Comments (e.g., if payments were made to you or to your institution)
	indicated in item #1 above).		
3	Royalties or licenses	☒ None	
4	Consulting fees	☒ None	
5	Payment or honoraria for lectures, presentations, speakers bureaus, manuscript writing or educational events	☒ None	
6	Payment for expert testimony	☒ None	
7	Support for attending meetings and/or travel	☒ None	
8	Patents planned, issued or pending	☒ None	
9	Participation on a Data Safety Monitoring Board or	☒ None	

		Name all entities with whom you have this relationship or indicate none (add rows as needed)	Specifications/Comments (e.g., if payments were made to you or to your institution)
	Advisory Board		
**10**	Leadership or fiduciary role in other board, society, committee or advocacy group, paid or unpaid	☒ **None**	
**11**	Stock or stock options	☒ **None**	
**12**	Receipt of equipment, materials, drugs, medical writing, gifts or other services	☒ **None**	
**13**	Other financial or non-financial interests	☒ **None**	

Please place an "X" next to the following statement to indicate your agreement:

☒ I certify that I have answered every question and have not altered the wording of any of the questions on this form.

ICMJE DISCLOSURE FORM

**Date:**	7/31/2026
**Your Name:**	Bianca Brijnath
**Manuscript Title:**	Clinician-interpreter collaboration in multilingual dementia assessments: Barriers, role boundaries, and clinician training needs
**Manuscript Number (if known):**	DADM-D-26-00213

In the interest of transparency, we ask you to disclose all relationships/activities/interests listed below that are related to the content of your manuscript. "Related" means any relation with for-profit or not-for-profit third parties whose interests may be affected by the content of the manuscript. Disclosure represents a commitment to transparency and does not necessarily indicate a bias. If you are in doubt about whether to list a relationship/activity/interest, it is preferable that you do so.

The author's relationships/activities/interests should be defined broadly. For example, if your manuscript pertains to the epidemiology of hypertension, you should declare all relationships with manufacturers of antihypertensive medication, even if that medication is not mentioned in the manuscript.

In item #1 below, report all support for the work reported in this manuscript without time limit. For all other items, the time frame for disclosure is the past 36 months.

		Name all entities with whom you have this relationship or indicate none (add rows as needed)	Specifications/Comments (e.g., if payments were made to you or to your institution)
**Time frame: Since the initial planning of the work**			
**1**	All support for the present manuscript (e.g., funding, provision of study materials, medical writing, article processing charges, etc.) **No time limit for this item.**	☐ **None**	
		National Health and Medical Research Council Australia	Grant funding to the National Ageing Research Institute (NARI)
		NAATI — National Accreditation Authority for Translators and Interpreters	Grant and in-kind funding to NARI
		AUSIT — Australian Institute of Interpreters and Translators	In-kind funding to the project
		Dementia Australia	In-kind funding to the project
		New South Wales Health Care Interpreting Service	In-kind funding to the project
		All Graduates Interpreting and Translation Services	Grant and in-kind funding to NARI
		Australian Government Department of Home Affairs Translation and Interpreting Service	In-kind funding to the project
		Australian Government Department of Health, Disability, and Ageing	Grant funding to NARI
		Televic	In-kind funding to the project
		Migrant and Refugee Health Partnership	In-kind funding to the project
**Time frame: past 36 months**			
**2**	Grants or contracts from any entity (if not	☒ **None**	

		Name all entities with whom you have this relationship or indicate none (add rows as needed)	Specifications/Comments (e.g., if payments were made to you or to your institution)
	indicated in item #1 above).		
3	Royalties or licenses	☒ None	
4	Consulting fees	☒ None	
5	Payment or honoraria for lectures, presentations, speakers bureaus, manuscript writing or educational events	☐ None	
		2M Language Services	Payment to the institution
6	Payment for expert testimony	☒ None	
7	Support for attending meetings and/or travel	☒ None	
8	Patents planned, issued or pending	☒ None	
9	Participation on a Data Safety Monitoring Board or	☒ None	

		Name all entities with whom you have this relationship or indicate none (add rows as needed)	Specifications/Comments (e.g., if payments were made to you or to your institution)
	Advisory Board		
**10**	Leadership or fiduciary role in other board, society, committee or advocacy group, paid or unpaid	☐ **None**	
		Participant on the governance committee of the Australian Multicultural Health Collaborative	Voluntary role, no payment
**11**	Stock or stock options	☒ **None**	
**12**	Receipt of equipment, materials, drugs, medical writing, gifts or other services	☒ **None**	
**13**	Other financial or non-financial interests	☒ **None**	

Please place an "X" next to the following statement to indicate your agreement:

☒ I certify that I have answered every question and have not altered the wording of any of the questions on this form.

ICMJE DISCLOSURE FORM

**Date:**	7/31/2026
**Your Name:**	Marina Cavuoto
**Manuscript Title:**	Clinician-interpreter collaboration in multilingual dementia assessments: Barriers, role boundaries, and clinician training needs
**Manuscript Number (if known):**	DADM-D-26-00213

In the interest of transparency, we ask you to disclose all relationships/activities/interests listed below that are related to the content of your manuscript. "Related" means any relation with for-profit or not-for-profit third parties whose interests may be affected by the content of the manuscript. Disclosure represents a commitment to transparency and does not necessarily indicate a bias. If you are in doubt about whether to list a relationship/activity/interest, it is preferable that you do so.

The author's relationships/activities/interests should be defined broadly. For example, if your manuscript pertains to the epidemiology of hypertension, you should declare all relationships with manufacturers of antihypertensive medication, even if that medication is not mentioned in the manuscript.

In item #1 below, report all support for the work reported in this manuscript without time limit. For all other items, the time frame for disclosure is the past 36 months.

		Name all entities with whom you have this relationship or indicate none (add rows as needed)	Specifications/Comments (e.g., if payments were made to you or to your institution)
**Time frame: Since the initial planning of the work**			
**1**	All support for the present manuscript (e.g., funding, provision of study materials, medical writing, article processing charges, etc.) **No time limit for this item.**	☐ **None**	
		National Health and Medical Research Council Australia	Grant funding to the National Ageing Research Institute (NARI)
		NAATI — National Accreditation Authority for Translators and Interpreters	Grant and in-kind funding to NARI
		AUSIT — Australian Institute of Interpreters and Translators	In-kind funding to the project
		Dementia Australia	In-kind funding to the project
		New South Wales Health Care Interpreting Service	In-kind funding to the project
		All Graduates Interpreting and Translation Services	Grant and in-kind funding to NARI
		Australian Government Department of Home Affairs Translation and Interpreting Service	In-kind funding to the project
		Australian Government Department of Health, Disability, and Ageing	Grant funding to NARI
		Televic	In-kind funding to the project
		Migrant and Refugee Health Partnership	In-kind funding to the project
**Time frame: past 36 months**			
**2**	Grants or contracts from any entity (if not	☒ **None**	

		Name all entities with whom you have this relationship or indicate none (add rows as needed)	Specifications/Comments (e.g., if payments were made to you or to your institution)
	indicated in item #1 above).		
3	Royalties or licenses	☒ None	
4	Consulting fees	☒ None	
5	Payment or honoraria for lectures, presentations, speakers bureaus, manuscript writing or educational events	☒ None	
6	Payment for expert testimony	☒ None	
7	Support for attending meetings and/or travel	☒ None	
8	Patents planned, issued or pending	☒ None	
9	Participation on a Data Safety Monitoring Board or	☒ None	

		Name all entities with whom you have this relationship or indicate none (add rows as needed)	Specifications/Comments (e.g., if payments were made to you or to your institution)
	Advisory Board		
10	Leadership or fiduciary role in other board, society, committee or advocacy group, paid or unpaid	☒ **None**	
11	Stock or stock options	☒ **None**	
12	Receipt of equipment, materials, drugs, medical writing, gifts or other services	☒ **None**	
13	Other financial or non-financial interests	☒ **None**	

Please place an "X" next to the following statement to indicate your agreement:

☒ I certify that I have answered every question and have not altered the wording of any of the questions on this form.

ICMJE DISCLOSURE FORM

**Date:**	7/31/2026
**Your Name:**	Nyssa Clarke
**Manuscript Title:**	Clinician-interpreter collaboration in multilingual dementia assessments: Barriers, role boundaries, and clinician training needs
**Manuscript Number (if known):**	DADM-D-26-00213

In the interest of transparency, we ask you to disclose all relationships/activities/interests listed below that are related to the content of your manuscript. "Related" means any relation with for-profit or not-for-profit third parties whose interests may be affected by the content of the manuscript. Disclosure represents a commitment to transparency and does not necessarily indicate a bias. If you are in doubt about whether to list a relationship/activity/interest, it is preferable that you do so.

The author's relationships/activities/interests should be defined broadly. For example, if your manuscript pertains to the epidemiology of hypertension, you should declare all relationships with manufacturers of antihypertensive medication, even if that medication is not mentioned in the manuscript.

In item #1 below, report all support for the work reported in this manuscript without time limit. For all other items, the time frame for disclosure is the past 36 months.

		**Name all entities with whom you have this relationship or indicate none (add rows as needed)**	**Specifications/Comments (e.g., if payments were made to you or to your institution)**
**Time frame: Since the initial planning of the work**			
**1**	All support for the present manuscript (e.g., funding, provision of study materials, medical writing, article processing charges, etc.) **No time limit for this item.**	☐ **None**	
		National Health and Medical Research Council Australia	Grant funding to the National Ageing Research Institute (NARI)
		NAATI — National Accreditation Authority for Translators and Interpreters	Grant and in-kind funding to NARI
		AUSIT — Australian Institute of Interpreters and Translators	In-kind funding to the project
		Dementia Australia	In-kind funding to the project
		New South Wales Health Care Interpreting Service	In-kind funding to the project
		All Graduates Interpreting and Translation Services	Grant and in-kind funding to NARI
		Australian Government Department of Home Affairs Translation and Interpreting Service	In-kind funding to the project
		Australian Government Department of Health, Disability, and Ageing	Grant funding to NARI
		Televic	In-kind funding to the project
		Migrant and Refugee Health Partnership	In-kind funding to the project
**Time frame: past 36 months**			
**2**	Grants or contracts from any entity (if not	☒ **None**	

		Name all entities with whom you have this relationship or indicate none (add rows as needed)	Specifications/Comments (e.g., if payments were made to you or to your institution)
	indicated in item #1 above).		
3	Royalties or licenses	☒ None	
4	Consulting fees	☒ None	
5	Payment or honoraria for lectures, presentations, speakers bureaus, manuscript writing or educational events	☒ None	
6	Payment for expert testimony	☒ None	
7	Support for attending meetings and/or travel	☒ None	
8	Patents planned, issued or pending	☒ None	
9	Participation on a Data Safety Monitoring Board or	☒ None	

		Name all entities with whom you have this relationship or indicate none (add rows as needed)	Specifications/Comments (e.g., if payments were made to you or to your institution)
	Advisory Board		
**10**	Leadership or fiduciary role in other board, society, committee or advocacy group, paid or unpaid	☒ **None**	
**11**	Stock or stock options	☒ **None**	
**12**	Receipt of equipment, materials, drugs, medical writing, gifts or other services	☒ **None**	
**13**	Other financial or non-financial interests	☒ **None**	

Please place an “X” next to the following statement to indicate your agreement:

☒ I certify that I have answered every question and have not altered the wording of any of the questions on this form.

ICMJE DISCLOSURE FORM

**Date:**	7/31/2026
**Your Name:**	Joanne Enticott
**Manuscript Title:**	Clinician-interpreter collaboration in multilingual dementia assessments: Barriers, role boundaries, and clinician training needs
**Manuscript Number (if known):**	DADM-D-26-00213

In the interest of transparency, we ask you to disclose all relationships/activities/interests listed below that are related to the content of your manuscript. "Related" means any relation with for-profit or not-for-profit third parties whose interests may be affected by the content of the manuscript. Disclosure represents a commitment to transparency and does not necessarily indicate a bias. If you are in doubt about whether to list a relationship/activity/interest, it is preferable that you do so.

The author's relationships/activities/interests should be defined broadly. For example, if your manuscript pertains to the epidemiology of hypertension, you should declare all relationships with manufacturers of antihypertensive medication, even if that medication is not mentioned in the manuscript.

In item #1 below, report all support for the work reported in this manuscript without time limit. For all other items, the time frame for disclosure is the past 36 months.

		**Name all entities with whom you have this relationship or indicate none (add rows as needed)**	**Specifications/Comments (e.g., if payments were made to you or to your institution)**
**Time frame: Since the initial planning of the work**			
**1**	All support for the present manuscript (e.g., funding, provision of study materials, medical writing, article processing charges, etc.) **No time limit for this item.**	☐ **None**	
		National Health and Medical Research Council Australia	Grant funding to the National Ageing Research Institute (NARI)
		NAATI — National Accreditation Authority for Translators and Interpreters	Grant and in-kind funding to NARI
		AUSIT — Australian Institute of Interpreters and Translators	In-kind funding to the project
		Dementia Australia	In-kind funding to the project
		New South Wales Health Care Interpreting Service	In-kind funding to the project
		All Graduates Interpreting and Translation Services	Grant and in-kind funding to NARI
		Australian Government Department of Home Affairs Translation and Interpreting Service	In-kind funding to the project
		Australian Government Department of Health, Disability, and Ageing	Grant funding to NARI
		Televic	In-kind funding to the project
		Migrant and Refugee Health Partnership	In-kind funding to the project
**Time frame: past 36 months**			
**2**	Grants or contracts from any entity (if not	☒ **None**	

		Name all entities with whom you have this relationship or indicate none (add rows as needed)	Specifications/Comments (e.g., if payments were made to you or to your institution)
	indicated in item #1 above).		
3	Royalties or licenses	☒ None	
4	Consulting fees	☒ None	
5	Payment or honoraria for lectures, presentations, speakers bureaus, manuscript writing or educational events	☒ None	
6	Payment for expert testimony	☒ None	
7	Support for attending meetings and/or travel	☒ None	
8	Patents planned, issued or pending	☒ None	
9	Participation on a Data Safety Monitoring Board or	☒ None	

		Name all entities with whom you have this relationship or indicate none (add rows as needed)	Specifications/Comments (e.g., if payments were made to you or to your institution)
	Advisory Board		
**10**	Leadership or fiduciary role in other board, society, committee or advocacy group, paid or unpaid	☒ **None**	
**11**	Stock or stock options	☒ **None**	
**12**	Receipt of equipment, materials, drugs, medical writing, gifts or other services	☒ **None**	
**13**	Other financial or non-financial interests	☒ **None**	

Please place an "X" next to the following statement to indicate your agreement:

☒ I certify that I have answered every question and have not altered the wording of any of the questions on this form.

ICMJE DISCLOSURE FORM

Date: 7/31/2026

Your Name: Andrew Simon Gilbert

Manuscript Title: Clinician-interpreter collaboration in multilingual dementia assessments: Barriers, role boundaries, and clinician training needs

Manuscript Number (if known): DADM-D-26-00213

In the interest of transparency, we ask you to disclose all relationships/activities/interests listed below that are related to the content of your manuscript. "Related" means any relation with for-profit or not-for-profit third parties whose interests may be affected by the content of the manuscript. Disclosure represents a commitment to transparency and does not necessarily indicate a bias. If you are in doubt about whether to list a relationship/activity/interest, it is preferable that you do so.

The author's relationships/activities/interests should be defined broadly. For example, if your manuscript pertains to the epidemiology of hypertension, you should declare all relationships with manufacturers of antihypertensive medication, even if that medication is not mentioned in the manuscript.

In item #1 below, report all support for the work reported in this manuscript without time limit. For all other items, the time frame for disclosure is the past 36 months.

		Name all entities with whom you have this relationship or indicate none (add rows as needed)	Specifications/Comments (e.g., if payments were made to you or to your institution)
**Time frame: Since the initial planning of the work**			
**1**	All support for the present manuscript (e.g., funding, provision of study materials, medical writing, article processing charges, etc.) **No time limit for this item.**	☐ **None**	
		National Health and Medical Research Council Australia	Grant funding to the National Ageing Research Institute (NARI)
		NAATI — National Accreditation Authority for Translators and Interpreters	Grant and in-kind funding to NARI
		AUSIT — Australian Institute of Interpreters and Translators	In-kind funding to the project
		Dementia Australia	In-kind funding to the project
		New South Wales Health Care Interpreting Service	In-kind funding to the project
		All Graduates Interpreting and Translation Services	Grant and in-kind funding to NARI
		Australian Government Department of Home Affairs Translation and Interpreting Service	In-kind funding to the project
		Australian Government Department of Health, Disability, and Ageing	Grant funding to NARI
		Televic	In-kind funding to the project
		Migrant and Refugee Health Partnership	In-kind funding to the project
**Time frame: past 36 months**			
**2**	Grants or contracts from any entity (if not	☒ **None**	

		Name all entities with whom you have this relationship or indicate none (add rows as needed)	Specifications/Comments (e.g., if payments were made to you or to your institution)
	indicated in item #1 above).		
3	Royalties or licenses	☒ None	
4	Consulting fees	☒ None	
5	Payment or honoraria for lectures, presentations, speakers bureaus, manuscript writing or educational events	☒ None	
6	Payment for expert testimony	☒ None	
7	Support for attending meetings and/or travel	☒ None	
8	Patents planned, issued or pending	☒ None	
9	Participation on a Data Safety Monitoring Board or	☒ None	

		Name all entities with whom you have this relationship or indicate none (add rows as needed)	Specifications/Comments (e.g., if payments were made to you or to your institution)
	Advisory Board		
**10**	Leadership or fiduciary role in other board, society, committee or advocacy group, paid or unpaid	☒ **None**	
**11**	Stock or stock options	☒ **None**	
**12**	Receipt of equipment, materials, drugs, medical writing, gifts or other services	☒ **None**	
**13**	Other financial or non-financial interests	☒ **None**	

Please place an "X" next to the following statement to indicate your agreement:

☒ I certify that I have answered every question and have not altered the wording of any of the questions on this form.

ICMJE DISCLOSURE FORM

Date: 7/31/2026

Your Name: Erika Gonzalez Garcia

Manuscript Title: Clinician-interpreter collaboration in multilingual dementia assessments: Barriers, role boundaries, and clinician training needs

Manuscript Number (if known): DADM-D-26-00213

In the interest of transparency, we ask you to disclose all relationships/activities/interests listed below that are related to the content of your manuscript. "Related" means any relation with for-profit or not-for-profit third parties whose interests may be affected by the content of the manuscript. Disclosure represents a commitment to transparency and does not necessarily indicate a bias. If you are in doubt about whether to list a relationship/activity/interest, it is preferable that you do so.

The author's relationships/activities/interests should be defined broadly. For example, if your manuscript pertains to the epidemiology of hypertension, you should declare all relationships with manufacturers of antihypertensive medication, even if that medication is not mentioned in the manuscript.

In item #1 below, report all support for the work reported in this manuscript without time limit. For all other items, the time frame for disclosure is the past 36 months.

		Name all entities with whom you have this relationship or indicate none (add rows as needed)	Specifications/Comments (e.g., if payments were made to you or to your institution)
**Time frame: Since the initial planning of the work**			
**1**	All support for the present manuscript (e.g., funding, provision of study materials, medical writing, article processing charges, etc.) **No time limit for this item.**	☐ **None**	
		National Health and Medical Research Council Australia	Grant funding to the National Ageing Research Institute (NARI)
		NAATI — National Accreditation Authority for Translators and Interpreters	Grant and in-kind funding to NARI
		AUSIT — Australian Institute of Interpreters and Translators	In-kind funding to the project
		Dementia Australia	In-kind funding to the project
		New South Wales Health Care Interpreting Service	In-kind funding to the project
		All Graduates Interpreting and Translation Services	Grant and in-kind funding to NARI
		Australian Government Department of Home Affairs Translation and Interpreting Service	In-kind funding to the project
		Australian Government Department of Health, Disability, and Ageing	Grant funding to NARI
		Televic	In-kind funding to the project
		Migrant and Refugee Health Partnership	In-kind funding to the project
**Time frame: past 36 months**			
**2**	Grants or contracts from any entity (if not	☒ **None**	

		Name all entities with whom you have this relationship or indicate none (add rows as needed)	Specifications/Comments (e.g., if payments were made to you or to your institution)
	indicated in item #1 above).		
3	Royalties or licenses	☒ None	
4	Consulting fees	☒ None	
5	Payment or honoraria for lectures, presentations, speakers bureaus, manuscript writing or educational events	☒ None	
6	Payment for expert testimony	☒ None	
7	Support for attending meetings and/or travel	☒ None	
8	Patents planned, issued or pending	☒ None	
9	Participation on a Data Safety Monitoring Board or	☒ None	

		Name all entities with whom you have this relationship or indicate none (add rows as needed)	Specifications/Comments (e.g., if payments were made to you or to your institution)
	Advisory Board		
**10**	Leadership or fiduciary role in other board, society, committee or advocacy group, paid or unpaid	☐ **None**	
		Past president of AUSIT	unpaid
**11**	Stock or stock options	☒ **None**	
**12**	Receipt of equipment, materials, drugs, medical writing, gifts or other services	☒ **None**	
**13**	Other financial or non-financial interests	☐ **None**	
		Works as an interpreter	Payment made to me

Please place an "X" next to the following statement to indicate your agreement:

☒ I certify that I have answered every question and have not altered the wording of any of the questions on this form.

ICMJE DISCLOSURE FORM

Date: 7/31/2026

Your Name: Jim Hlavac

Manuscript Title: Clinician-interpreter collaboration in multilingual dementia assessments: Barriers, role boundaries, and clinician training needs

Manuscript Number (if known): DADM-D-26-00213

In the interest of transparency, we ask you to disclose all relationships/activities/interests listed below that are related to the content of your manuscript. "Related" means any relation with for-profit or not-for-profit third parties whose interests may be affected by the content of the manuscript. Disclosure represents a commitment to transparency and does not necessarily indicate a bias. If you are in doubt about whether to list a relationship/activity/interest, it is preferable that you do so.

The author's relationships/activities/interests should be defined broadly. For example, if your manuscript pertains to the epidemiology of hypertension, you should declare all relationships with manufacturers of antihypertensive medication, even if that medication is not mentioned in the manuscript.

In item #1 below, report all support for the work reported in this manuscript without time limit. For all other items, the time frame for disclosure is the past 36 months.

		Name all entities with whom you have this relationship or indicate none (add rows as needed)	Specifications/Comments (e.g., if payments were made to you or to your institution)
**Time frame: Since the initial planning of the work**			
**1**	All support for the present manuscript (e.g., funding, provision of study materials, medical writing, article processing charges, etc.) **No time limit for this item.**	☐ **None**	
		National Health and Medical Research Council Australia	Grant funding to the National Ageing Research Institute (NARI)
		NAATI — National Accreditation Authority for Translators and Interpreters	Grant and in-kind funding to NARI
		AUSIT — Australian Institute of Interpreters and Translators	In-kind funding to the project
		Dementia Australia	In-kind funding to the project
		New South Wales Health Care Interpreting Service	In-kind funding to the project
		All Graduates Interpreting and Translation Services	Grant and in-kind funding to NARI
		Australian Government Department of Home Affairs Translation and Interpreting Service	In-kind funding to the project
		Australian Government Department of Health, Disability, and Ageing	Grant funding to NARI
		Televic	In-kind funding to the project
		Migrant and Refugee Health Partnership	In-kind funding to the project
**Time frame: past 36 months**			
**2**	Grants or contracts from any entity (if not	☒ **None**	

		Name all entities with whom you have this relationship or indicate none (add rows as needed)	Specifications/Comments (e.g., if payments were made to you or to your institution)
	indicated in item #1 above).		
3	Royalties or licenses	☒ None	
4	Consulting fees	☒ None	
5	Payment or honoraria for lectures, presentations, speakers bureaus, manuscript writing or educational events	☒ None	
6	Payment for expert testimony	☒ None	
7	Support for attending meetings and/or travel	☒ None	
8	Patents planned, issued or pending	☒ None	
9	Participation on a Data Safety Monitoring Board or	☒ None	

		Name all entities with whom you have this relationship or indicate none (add rows as needed)	Specifications/Comments (e.g., if payments were made to you or to your institution)
	Advisory Board		
**10**	Leadership or fiduciary role in other board, society, committee or advocacy group, paid or unpaid	☒ **None**	
**11**	Stock or stock options	☒ **None**	
**12**	Receipt of equipment, materials, drugs, medical writing, gifts or other services	☒ **None**	
**13**	Other financial or non-financial interests	☐ **None**	
		Works as an interpreter	Payment made to me

Please place an "X" next to the following statement to indicate your agreement:

☒ I certify that I have answered every question and have not altered the wording of any of the questions on this form.

ICMJE DISCLOSURE FORM

**Date:**	7/31/2026
**Your Name:**	Kerry Hwang
**Manuscript Title:**	Clinician-interpreter collaboration in multilingual dementia assessments: Barriers, role boundaries, and clinician training needs
**Manuscript Number (if known):**	DADM-D-26-00213

In the interest of transparency, we ask you to disclose all relationships/activities/interests listed below that are related to the content of your manuscript. "Related" means any relation with for-profit or not-for-profit third parties whose interests may be affected by the content of the manuscript. Disclosure represents a commitment to transparency and does not necessarily indicate a bias. If you are in doubt about whether to list a relationship/activity/interest, it is preferable that you do so.

The author's relationships/activities/interests should be defined broadly. For example, if your manuscript pertains to the epidemiology of hypertension, you should declare all relationships with manufacturers of antihypertensive medication, even if that medication is not mentioned in the manuscript.

In item #1 below, report all support for the work reported in this manuscript without time limit. For all other items, the time frame for disclosure is the past 36 months.

		**Name all entities with whom you have this relationship or indicate none (add rows as needed)**	**Specifications/Comments (e.g., if payments were made to you or to your institution)**
**Time frame: Since the initial planning of the work**			
**1**	All support for the present manuscript (e.g., funding, provision of study materials, medical writing, article processing charges, etc.) **No time limit for this item.**	☐ **None**	
		National Health and Medical Research Council Australia	Grant funding to the National Ageing Research Institute (NARI)
		NAATI — National Accreditation Authority for Translators and Interpreters	Grant and in-kind funding to NARI
		AUSIT — Australian Institute of Interpreters and Translators	In-kind funding to the project
		Dementia Australia	In-kind funding to the project
		New South Wales Health Care Interpreting Service	In-kind funding to the project
		All Graduates Interpreting and Translation Services	Grant and in-kind funding to NARI
		Australian Government Department of Home Affairs Translation and Interpreting Service	In-kind funding to the project
		Australian Government Department of Health, Disability, and Ageing	Grant funding to NARI
		Televic	In-kind funding to the project
		Migrant and Refugee Health Partnership	In-kind funding to the project
**Time frame: past 36 months**			
**2**	Grants or contracts from any entity (if not	☒ **None**	

		Name all entities with whom you have this relationship or indicate none (add rows as needed)	Specifications/Comments (e.g., if payments were made to you or to your institution)
	indicated in item #1 above).		
3	Royalties or licenses	☒ None	
4	Consulting fees	☒ None	
5	Payment or honoraria for lectures, presentations, speakers bureaus, manuscript writing or educational events	☒ None	
6	Payment for expert testimony	☒ None	
7	Support for attending meetings and/or travel	☒ None	
8	Patents planned, issued or pending	☒ None	
9	Participation on a Data Safety Monitoring Board or	☒ None	

		Name all entities with whom you have this relationship or indicate none (add rows as needed)	Specifications/Comments (e.g., if payments were made to you or to your institution)
	Advisory Board		
**10**	Leadership or fiduciary role in other board, society, committee or advocacy group, paid or unpaid	☒ **None**	
**11**	Stock or stock options	☒ **None**	
**12**	Receipt of equipment, materials, drugs, medical writing, gifts or other services	☒ **None**	
**13**	Other financial or non-financial interests	☒ **None**	

Please place an "X" next to the following statement to indicate your agreement:

☒ I certify that I have answered every question and have not altered the wording of any of the questions on this form.

ICMJE DISCLOSURE FORM

**Date:**	7/31/2026
**Your Name:**	Xiaoping Lin
**Manuscript Title:**	Clinician-interpreter collaboration in multilingual dementia assessments: Barriers, role boundaries, and clinician training needs
**Manuscript Number (if known):**	DADM-D-26-00213

In the interest of transparency, we ask you to disclose all relationships/activities/interests listed below that are related to the content of your manuscript. "Related" means any relation with for-profit or not-for-profit third parties whose interests may be affected by the content of the manuscript. Disclosure represents a commitment to transparency and does not necessarily indicate a bias. If you are in doubt about whether to list a relationship/activity/interest, it is preferable that you do so.

The author's relationships/activities/interests should be defined broadly. For example, if your manuscript pertains to the epidemiology of hypertension, you should declare all relationships with manufacturers of antihypertensive medication, even if that medication is not mentioned in the manuscript.

In item #1 below, report all support for the work reported in this manuscript without time limit. For all other items, the time frame for disclosure is the past 36 months.

		Name all entities with whom you have this relationship or indicate none (add rows as needed)	Specifications/Comments (e.g., if payments were made to you or to your institution)
**Time frame: Since the initial planning of the work**			
**1**	All support for the present manuscript (e.g., funding, provision of study materials, medical writing, article processing charges, etc.) **No time limit for this item.**	☐ **None**	
		National Health and Medical Research Council Australia	Grant funding to the National Ageing Research Institute (NARI)
		NAATI — National Accreditation Authority for Translators and Interpreters	Grant and in-kind funding to NARI
		AUSIT — Australian Institute of Interpreters and Translators	In-kind funding to the project
		Dementia Australia	In-kind funding to the project
		New South Wales Health Care Interpreting Service	In-kind funding to the project
		All Graduates Interpreting and Translation Services	Grant and in-kind funding to NARI
		Australian Government Department of Home Affairs Translation and Interpreting Service	In-kind funding to the project
		Australian Government Department of Health, Disability, and Ageing	Grant funding to NARI
		Televic	In-kind funding to the project
		Migrant and Refugee Health Partnership	In-kind funding to the project
**Time frame: past 36 months**			
**2**	Grants or contracts from any entity (if not	☒ **None**	

		Name all entities with whom you have this relationship or indicate none (add rows as needed)	Specifications/Comments (e.g., if payments were made to you or to your institution)
	indicated in item #1 above).		
3	Royalties or licenses	☒ None	
4	Consulting fees	☒ None	
5	Payment or honoraria for lectures, presentations, speakers bureaus, manuscript writing or educational events	☒ None	
6	Payment for expert testimony	☒ None	
7	Support for attending meetings and/or travel	☒ None	
8	Patents planned, issued or pending	☒ None	
9	Participation on a Data Safety Monitoring Board or	☒ None	

		Name all entities with whom you have this relationship or indicate none (add rows as needed)	Specifications/Comments (e.g., if payments were made to you or to your institution)
	Advisory Board		
10	Leadership or fiduciary role in other board, society, committee or advocacy group, paid or unpaid	☒ None	
11	Stock or stock options	☒ None	
12	Receipt of equipment, materials, drugs, medical writing, gifts or other services	☒ None	
13	Other financial or non-financial interests	☒ None	

Please place an "X" next to the following statement to indicate your agreement:

☒ I certify that I have answered every question and have not altered the wording of any of the questions on this form.

ICMJE DISCLOSURE FORM

Date: 7/31/2026

Your Name: Dina LoGiudice

Manuscript Title: Clinician-interpreter collaboration in multilingual dementia assessments: Barriers, role boundaries, and clinician training needs

Manuscript Number (if known): DADM-D-26-00213

In the interest of transparency, we ask you to disclose all relationships/activities/interests listed below that are related to the content of your manuscript. "Related" means any relation with for-profit or not-for-profit third parties whose interests may be affected by the content of the manuscript. Disclosure represents a commitment to transparency and does not necessarily indicate a bias. If you are in doubt about whether to list a relationship/activity/interest, it is preferable that you do so.

The author's relationships/activities/interests should be defined broadly. For example, if your manuscript pertains to the epidemiology of hypertension, you should declare all relationships with manufacturers of antihypertensive medication, even if that medication is not mentioned in the manuscript.

In item #1 below, report all support for the work reported in this manuscript without time limit. For all other items, the time frame for disclosure is the past 36 months.

		Name all entities with whom you have this relationship or indicate none (add rows as needed)	Specifications/Comments (e.g., if payments were made to you or to your institution)
**Time frame: Since the initial planning of the work**			
**1**	All support for the present manuscript (e.g., funding, provision of study materials, medical writing, article processing charges, etc.) **No time limit for this item.**	☐ **None**	
		National Health and Medical Research Council Australia	Grant funding to the National Ageing Research Institute (NARI)
		NAATI — National Accreditation Authority for Translators and Interpreters	Grant and in-kind funding to NARI
		AUSIT — Australian Institute of Interpreters and Translators	In-kind funding to the project
		Dementia Australia	In-kind funding to the project
		New South Wales Health Care Interpreting Service	In-kind funding to the project
		All Graduates Interpreting and Translation Services	Grant and in-kind funding to NARI
		Australian Government Department of Home Affairs Translation and Interpreting Service	In-kind funding to the project
		Australian Government Department of Health, Disability, and Ageing	Grant funding to NARI
		Televic	In-kind funding to the project
		Migrant and Refugee Health Partnership	In-kind funding to the project
**Time frame: past 36 months**			
**2**	Grants or contracts from any entity (if not	☒ **None**	

		Name all entities with whom you have this relationship or indicate none (add rows as needed)	Specifications/Comments (e.g., if payments were made to you or to your institution)
	indicated in item #1 above).		
3	Royalties or licenses	☒ None	
4	Consulting fees	☒ None	
5	Payment or honoraria for lectures, presentations, speakers bureaus, manuscript writing or educational events	☒ None	
6	Payment for expert testimony	☒ None	
7	Support for attending meetings and/or travel	☒ None	
8	Patents planned, issued or pending	☒ None	
9	Participation on a Data Safety Monitoring Board or	☒ None	

		Name all entities with whom you have this relationship or indicate none (add rows as needed)	Specifications/Comments (e.g., if payments were made to you or to your institution)
	Advisory Board		
**10**	Leadership or fiduciary role in other board, society, committee or advocacy group, paid or unpaid	☒ **None**	
**11**	Stock or stock options	☒ **None**	
**12**	Receipt of equipment, materials, drugs, medical writing, gifts or other services	☒ **None**	
**13**	Other financial or non-financial interests	☒ **None**	

Please place an "X" next to the following statement to indicate your agreement:

☒ I certify that I have answered every question and have not altered the wording of any of the questions on this form.

ICMJE DISCLOSURE FORM

**Date:**	7/31/2026
**Your Name:**	Lee-Fay Low
**Manuscript Title:**	Clinician-interpreter collaboration in multilingual dementia assessments: Barriers, role boundaries, and clinician training needs
**Manuscript Number (if known):**	DADM-D-26-00213

In the interest of transparency, we ask you to disclose all relationships/activities/interests listed below that are related to the content of your manuscript. "Related" means any relation with for-profit or not-for-profit third parties whose interests may be affected by the content of the manuscript. Disclosure represents a commitment to transparency and does not necessarily indicate a bias. If you are in doubt about whether to list a relationship/activity/interest, it is preferable that you do so.

The author's relationships/activities/interests should be defined broadly. For example, if your manuscript pertains to the epidemiology of hypertension, you should declare all relationships with manufacturers of antihypertensive medication, even if that medication is not mentioned in the manuscript.

In item #1 below, report all support for the work reported in this manuscript without time limit. For all other items, the time frame for disclosure is the past 36 months.

		**Name all entities with whom you have this relationship or indicate none (add rows as needed)**	**Specifications/Comments (e.g., if payments were made to you or to your institution)**
		**Time frame: Since the initial planning of the work**	
**1**	All support for the present manuscript (e.g., funding, provision of study materials, medical writing, article processing charges, etc.) **No time limit for this item.**	☐ **None**	
		National Health and Medical Research Council Australia	Grant funding to the National Ageing Research Institute (NARI)
		NAATI — National Accreditation Authority for Translators and Interpreters	Grant and in-kind funding to NARI
		AUSIT — Australian Institute of Interpreters and Translators	In-kind funding to the project
		Dementia Australia	In-kind funding to the project
		New South Wales Health Care Interpreting Service	In-kind funding to the project
		All Graduates Interpreting and Translation Services	Grant and in-kind funding to NARI
		Australian Government Department of Home Affairs Translation and Interpreting Service	In-kind funding to the project
		Australian Government Department of Health, Disability, and Ageing	Grant funding to NARI
		Televic	In-kind funding to the project
		Migrant and Refugee Health Partnership	In-kind funding to the project
		**Time frame: past 36 months**	
**2**	Grants or contracts from any entity (if not	☒ **None**	

		Name all entities with whom you have this relationship or indicate none (add rows as needed)	Specifications/Comments (e.g., if payments were made to you or to your institution)
	indicated in item #1 above).		
3	Royalties or licenses	☒ None	
4	Consulting fees	☒ None	
5	Payment or honoraria for lectures, presentations, speakers bureaus, manuscript writing or educational events	☒ None	
6	Payment for expert testimony	☒ None	
7	Support for attending meetings and/or travel	☒ None	
8	Patents planned, issued or pending	☒ None	
9	Participation on a Data Safety Monitoring Board or	☒ None	

		Name all entities with whom you have this relationship or indicate none (add rows as needed)	Specifications/Comments (e.g., if payments were made to you or to your institution)
	Advisory Board		
**10**	Leadership or fiduciary role in other board, society, committee or advocacy group, paid or unpaid	☒ **None**	
**11**	Stock or stock options	☒ **None**	
**12**	Receipt of equipment, materials, drugs, medical writing, gifts or other services	☒ **None**	
**13**	Other financial or non-financial interests	☒ **None**	

Please place an "X" next to the following statement to indicate your agreement:

☒ I certify that I have answered every question and have not altered the wording of any of the questions on this form.

ICMJE DISCLOSURE FORM

**Date:**	7/31/2026
**Your Name:**	Simona Markusevska
**Manuscript Title:**	Clinician-interpreter collaboration in multilingual dementia assessments: Barriers, role boundaries, and clinician training needs
**Manuscript Number (if known):**	DADM-D-26-00213

In the interest of transparency, we ask you to disclose all relationships/activities/interests listed below that are related to the content of your manuscript. "Related" means any relation with for-profit or not-for-profit third parties whose interests may be affected by the content of the manuscript. Disclosure represents a commitment to transparency and does not necessarily indicate a bias. If you are in doubt about whether to list a relationship/activity/interest, it is preferable that you do so.

The author's relationships/activities/interests should be defined broadly. For example, if your manuscript pertains to the epidemiology of hypertension, you should declare all relationships with manufacturers of antihypertensive medication, even if that medication is not mentioned in the manuscript.

In item #1 below, report all support for the work reported in this manuscript without time limit. For all other items, the time frame for disclosure is the past 36 months.

		**Name all entities with whom you have this relationship or indicate none (add rows as needed)**	**Specifications/Comments (e.g., if payments were made to you or to your institution)**
**Time frame: Since the initial planning of the work**			
**1**	All support for the present manuscript (e.g., funding, provision of study materials, medical writing, article processing charges, etc.) **No time limit for this item.**	☐ **None**	
		National Health and Medical Research Council Australia	Grant funding to the National Ageing Research Institute (NARI)
		NAATI — National Accreditation Authority for Translators and Interpreters	Grant and in-kind funding to NARI
		AUSIT — Australian Institute of Interpreters and Translators	In-kind funding to the project
		Dementia Australia	In-kind funding to the project
		New South Wales Health Care Interpreting Service	In-kind funding to the project
		All Graduates Interpreting and Translation Services	Grant and in-kind funding to NARI
		Australian Government Department of Home Affairs Translation and Interpreting Service	In-kind funding to the project
		Australian Government Department of Health, Disability, and Ageing	Grant funding to NARI
		Televic	In-kind funding to the project
		Migrant and Refugee Health Partnership	In-kind funding to the project
**Time frame: past 36 months**			
**2**	Grants or contracts from any entity (if not	☒ **None**	

		Name all entities with whom you have this relationship or indicate none (add rows as needed)	Specifications/Comments (e.g., if payments were made to you or to your institution)
	indicated in item #1 above).		
3	Royalties or licenses	☒ None	
4	Consulting fees	☒ None	
5	Payment or honoraria for lectures, presentations, speakers bureaus, manuscript writing or educational events	☒ None	
6	Payment for expert testimony	☒ None	
7	Support for attending meetings and/or travel	☒ None	
8	Patents planned, issued or pending	☒ None	
9	Participation on a Data Safety Monitoring Board or	☒ None	

		Name all entities with whom you have this relationship or indicate none (add rows as needed)	Specifications/Comments (e.g., if payments were made to you or to your institution)
	Advisory Board		
**10**	Leadership or fiduciary role in other board, society, committee or advocacy group, paid or unpaid	☒ **None**	
**11**	Stock or stock options	☒ **None**	
**12**	Receipt of equipment, materials, drugs, medical writing, gifts or other services	☒ **None**	
**13**	Other financial or non-financial interests	☒ **None**	

Please place an "X" next to the following statement to indicate your agreement:

☒ I certify that I have answered every question and have not altered the wording of any of the questions on this form.

ICMJE DISCLOSURE FORM

**Date:**	7/31/2026
**Your Name:**	Jennifer White
**Manuscript Title:**	Clinician-interpreter collaboration in multilingual dementia assessments: Barriers, role boundaries, and clinician training needs
**Manuscript Number (if known):**	DADM-D-26-00213

In the interest of transparency, we ask you to disclose all relationships/activities/interests listed below that are related to the content of your manuscript. "Related" means any relation with for-profit or not-for-profit third parties whose interests may be affected by the content of the manuscript. Disclosure represents a commitment to transparency and does not necessarily indicate a bias. If you are in doubt about whether to list a relationship/activity/interest, it is preferable that you do so.

The author's relationships/activities/interests should be defined broadly. For example, if your manuscript pertains to the epidemiology of hypertension, you should declare all relationships with manufacturers of antihypertensive medication, even if that medication is not mentioned in the manuscript.

In item #1 below, report all support for the work reported in this manuscript without time limit. For all other items, the time frame for disclosure is the past 36 months.

		**Name all entities with whom you have this relationship or indicate none (add rows as needed)**	**Specifications/Comments (e.g., if payments were made to you or to your institution)**
		**Time frame: Since the initial planning of the work**	
**1**	All support for the present manuscript (e.g., funding, provision of study materials, medical writing, article processing charges, etc.) **No time limit for this item.**	☐ **None**	
		National Health and Medical Research Council Australia	Grant funding to the National Ageing Research Institute (NARI)
		NAATI — National Accreditation Authority for Translators and Interpreters	Grant and in-kind funding to NARI
		AUSIT — Australian Institute of Interpreters and Translators	In-kind funding to the project
		Dementia Australia	In-kind funding to the project
		New South Wales Health Care Interpreting Service	In-kind funding to the project
		All Graduates Interpreting and Translation Services	Grant and in-kind funding to NARI
		Australian Government Department of Home Affairs Translation and Interpreting Service	In-kind funding to the project
		Australian Government Department of Health, Disability, and Ageing	Grant funding to NARI
		Televic	In-kind funding to the project
		Migrant and Refugee Health Partnership	In-kind funding to the project
		**Time frame: past 36 months**	
**2**	Grants or contracts from any entity (if not	☒ **None**	

		Name all entities with whom you have this relationship or indicate none (add rows as needed)	Specifications/Comments (e.g., if payments were made to you or to your institution)
	indicated in item #1 above).		
3	Royalties or licenses	☒ None	
4	Consulting fees	☒ None	
5	Payment or honoraria for lectures, presentations, speakers bureaus, manuscript writing or educational events	☒ None	
6	Payment for expert testimony	☒ None	
7	Support for attending meetings and/or travel	☒ None	
8	Patents planned, issued or pending	☒ None	
9	Participation on a Data Safety Monitoring Board or	☒ None	

		Name all entities with whom you have this relationship or indicate none (add rows as needed)	Specifications/Comments (e.g., if payments were made to you or to your institution)
	Advisory Board		
**10**	Leadership or fiduciary role in other board, society, committee or advocacy group, paid or unpaid	☒ **None**	
**11**	Stock or stock options	☒ **None**	
**12**	Receipt of equipment, materials, drugs, medical writing, gifts or other services	☒ **None**	
**13**	Other financial or non-financial interests	☒ **None**	

Please place an "X" next to the following statement to indicate your agreement:

☒ I certify that I have answered every question and have not altered the wording of any of the questions on this form.

ICMJE DISCLOSURE FORM

**Date:**	7/31/2026
**Your Name:**	Robyn Woodward-Kron
**Manuscript Title:**	Clinician-interpreter collaboration in multilingual dementia assessments: Barriers, role boundaries, and clinician training needs
**Manuscript Number (if known):**	DADM-D-26-00213

In the interest of transparency, we ask you to disclose all relationships/activities/interests listed below that are related to the content of your manuscript. "Related" means any relation with for-profit or not-for-profit third parties whose interests may be affected by the content of the manuscript. Disclosure represents a commitment to transparency and does not necessarily indicate a bias. If you are in doubt about whether to list a relationship/activity/interest, it is preferable that you do so.

The author's relationships/activities/interests should be defined broadly. For example, if your manuscript pertains to the epidemiology of hypertension, you should declare all relationships with manufacturers of antihypertensive medication, even if that medication is not mentioned in the manuscript.

In item #1 below, report all support for the work reported in this manuscript without time limit. For all other items, the time frame for disclosure is the past 36 months.

		**Name all entities with whom you have this relationship or indicate none (add rows as needed)**	**Specifications/Comments (e.g., if payments were made to you or to your institution)**
**Time frame: Since the initial planning of the work**			
**1**	All support for the present manuscript (e.g., funding, provision of study materials, medical writing, article processing charges, etc.) **No time limit for this item.**	☐ **None**	
		National Health and Medical Research Council Australia	Grant funding to the National Ageing Research Institute (NARI)
		NAATI — National Accreditation Authority for Translators and Interpreters	Grant and in-kind funding to NARI
		AUSIT — Australian Institute of Interpreters and Translators	In-kind funding to the project
		Dementia Australia	In-kind funding to the project
		New South Wales Health Care Interpreting Service	In-kind funding to the project
		All Graduates Interpreting and Translation Services	Grant and in-kind funding to NARI
		Australian Government Department of Home Affairs Translation and Interpreting Service	In-kind funding to the project
		Australian Government Department of Health, Disability, and Ageing	Grant funding to NARI
		Televic	In-kind funding to the project
		Migrant and Refugee Health Partnership	In-kind funding to the project
**Time frame: past 36 months**			
**2**	Grants or contracts from any entity (if not	☒ **None**	

		Name all entities with whom you have this relationship or indicate none (add rows as needed)	Specifications/Comments (e.g., if payments were made to you or to your institution)
	indicated in item #1 above).		
3	Royalties or licenses	☒ None	
4	Consulting fees	☒ None	
5	Payment or honoraria for lectures, presentations, speakers bureaus, manuscript writing or educational events	☒ None	
6	Payment for expert testimony	☒ None	
7	Support for attending meetings and/or travel	☒ None	
8	Patents planned, issued or pending	☒ None	
9	Participation on a Data Safety Monitoring Board or	☒ None	

		Name all entities with whom you have this relationship or indicate none (add rows as needed)	Specifications/Comments (e.g., if payments were made to you or to your institution)
	Advisory Board		
**10**	Leadership or fiduciary role in other board, society, committee or advocacy group, paid or unpaid	☒ **None**	
**11**	Stock or stock options	☒ **None**	
**12**	Receipt of equipment, materials, drugs, medical writing, gifts or other services	☒ **None**	
**13**	Other financial or non-financial interests	☒ **None**	

Please place an "X" next to the following statement to indicate your agreement:

☒ I certify that I have answered every question and have not altered the wording of any of the questions on this form.
