# Supplementary material for: Clinician‐interpreter collaboration in multilingual dementia assessments: Barriers, role boundaries, and clinician training needs
Source: Alzheimers Dement (Amst). 2026 Oct 20;18(4):e70515. doi: 10.1002/dad2.70515 (PMC13683551; doi:10.1002/dad2.70515)
Supplement: Supplementary file 2 [file DAD2-18-e70515-s001.docx]

**Clinician-interpreter collaboration in multilingual dementia assessments: Barriers, role boundaries, and clinician training needs**

## Appendix file 2: Interview guides for clinicians and interpreters

**Interview - Clinicians**

| **PARTICIPANT DEMOGRAPHIC INFORMATION** | |
| --- | --- |
| Participant ID |  |
| Pseudonym |  |
| Gender |  |
| Professional role |  |
| Number of years working with PLWD |  |
| Other relevant comments |  |
| **INTERVIEW DETAILS** | |
| Date (dd/mm/yyyy) |  |
| Time commenced |  |
| Location |  |
| Interviewer |  |

**ADMINISTRATION OF THE INTERVIEW**

•	To be held at time and place of choice of participant

•	May be conducted online (Zoom or Skype), via telephone, or in person.

•	Ensure the interviewee and interviewer are sitting comfortably

•	Ensure all participants are clearly heard through the recording device.

•	The interview will be semi structured in that the researcher will use prompts to ask particular questions after which questions and answers will be open-ended.

**PREAMBLE**

**READ OUT** The purpose of the interview is to explore the impact that MINDSET training has had on interpreters working with people with dementia, and what could be done to improve its impact.

**INTERVIEW PROTOCOL**

1. Thank you for agreeing to be involved
2. Provide PICF
3. Allow time for PICF to be read
4. Explain project and PICF
5. Explain the purpose of the interview again and that the interview will be audio recorded and if consent gained for recording.
6. Seek consent
7. If consent gained, parties (interviewee, interviewer and witness, if required) sign consent form.
8. Researcher to state his/her name, date and confirm participants pseudonym for use of the interview. Outline consent has been made and ask again to confirm participant is happy to proceed.
9. Ask for any missing participant demographic information
10. Remind the participant that confidentiality and privacy can be assured where possible. No names will be transcribed from the audio recording so that it can remain de-identified.
11. All comments are valuable, there are no right or wrong answers.
12. The interview will be approximately 45 minutes. If a break is required, then the participant is encouraged to ask for one and this will be accommodated.
13. Commence interview.
14. Ask them to confirm for the audio tape that they:
    i) understand the interview process
    ii) understand they are free to withdraw at any time
    iii) give their consent for the interview to be recorded

**INTERVIEW QUESTIONS**

**Training impact**

1. How often do you use interpreters in your work?
2. How do you find working with interpreters, generally speaking?
   a. Is it something you’re confident doing?
   b. With people with dementia?
   c. For cognitive assessments?
3. Had you recognized a need for dementia-specific training for interpreters before hearing about MINDSET?
4. How did you first hear about the MINDSET training?
5. Do you know whether many interpreters you have used have received the training?

**Quality of interpreted communication**

1. Do you think the MINDSET training has affected the way interpreters communicate with your clients?
   a. Why/why not?
2. What specific things have you noticed that may have changed as a result of the MINDSET training?

*Possible prompts:*

i. Briefings/debriefings
ii. Rapport building
iii. Knowledge of cognitive assessment tools
iv. Knowledge of dementia
v. Use of terminology
vi. Sensitivity towards with people with dementia
vii. Professionalism
viii. Anything else?

3. How is that different to usual?
   a. How would you define ‘usual’?
   b. Is there anything that hasn’t changed which should?

**Improving MINDSET**

1. Is there anything that could be done to improve the impact of MINDSET on interpreters’ practice?
2. Would you recommend MINDSET to interpreters?
   a. Why/why not?
3. Would you say you are more satisfied with interpreters who have completed the training than those who have not?
   a. Why/why not?

**Interview - Interpreters**

| **PARTICIPANT DEMOGRAPHIC INFORMATION** | |
| --- | --- |
| Participant ID |  |
| Pseudonym |  |
| Gender |  |
| Professional role |  |
| Number of years interpreting |  |
| Other relevant comments |  |
| **INTERVIEW DETAILS** | |
| Date (dd/mm/yyyy) |  |
| Time commenced |  |
| Location |  |
| Interviewer |  |

**ADMINISTRATION OF THE INTERVIEW**

•	To be held at time and place of choice of participant

•	May be conducted online (Zoom or Skype), via telephone, or in person.

•	Ensure the interviewee and interviewer are sitting comfortably

•	Ensure all participants are clearly heard through the recording device.

•	The interview will be semi structured in that the researcher will use prompts to ask particular questions after which questions and answers will be open-ended.

**PREAMBLE**

**READ OUT** The purpose of the interview is to explore the impact that MINDSET training has had on interpreters working with people with dementia, and what could be done to improve its impact.

**INTERVIEW PROTOCOL**

- Thank you for agreeing to be involved
- Provide PICF
- Allow time for PICF to be read
- Explain project and PICF
- Explain the purpose of the interview again and that the interview will be audio recorded and if consent gained for recording.
- Seek consent
- If consent gained, parties (interviewee, interviewer and witness, if required) sign consent form.
- Researcher to state his/her name, date and confirm participants pseudonym for use of the interview. Outline consent has been made and ask again to confirm participant is happy to proceed.
- Ask for any missing participant demographic information
- Remind the participant that confidentiality and privacy can be assured where possible. No names will be transcribed from the audio recording so that it can remain de-identified.
- All comments are valuable, there are no right or wrong answers.
- The interview will be approximately 45 minutes. If a break is required, then the participant is encouraged to ask for one and this will be accommodated.
- Commence interview.
- Ask them to confirm for the audio tape that they:
  - understand the interview process
  - understand they are free to withdraw at any time
  - give their consent for the interview to be recorded

**INTERVIEW QUESTIONS**

**Training uptake**

1. How did you first hear about the MINDSET training?
2. What made you decide to do the training?
3. How important were the NAATI CPD points in motivating you to undertake it?
4. Had you recognized a need for dementia-specific training for interpreters before hearing about MINDSET?
5. What, if any, benefits do you see to doing the MINDSET training?

**Barriers and facilitators**

1. How was your experience in accessing the training?
   a. Did you encounter any issues accessing the training?
2. How long did it take you to do the training?
3. Once you accessed the training, did you encounter any problems completing it?
4. How was the online platform to use?
   a. How could it be improved?
5. Did the purpose of the training make sense?
   a. How could this be improved?
6. Were there any issues completing the assessment questions?
   a. How could this be improved?
7. Do you have any suggestions to improve the training?

**Impact on practice**

1. Is the MINDSET training something you have found useful in your professional work?
   a. Why/why not?
   b. Have you interpreted for a cognitive assessment since doing the training?
2. Can you tell me about a time that MINDSET has changed the way you work as an interpreter?
   a. What hasn’t changed?
3. Do you agree with the things you learned in the training?
   a. What did you agree with?
   b. Was there anything you disagreed with?
4. Would you say you follow advice from the training in your work?
   a. Why/why not?
   b. Is there any advice that’s difficult to follow?
5. Is there anything that could be done to improve the impact of MINDSET on interpreters’ practice?
6. Would you recommend MINDSET to other interpreters?
   a. Why/why not?
